# Editorial: The role of inflammasome in viral infection

**DOI:** 10.3389/fcimb.2023.1260013

**Published:** 2023-08-07

**Authors:** Pin Wan, Yaru Zhang, Pan Pan, Yongkui Li

**Affiliations:** ^1^ Wuhan Institute of Biomedical Sciences, School of Medicine, Jianghan University, Wuhan, China; ^2^ Foshan Institute of Medical Microbiology, Foshan, China; ^3^ Institute of Medical Microbiology, Jinan University, Guangzhou, China; ^4^ Laboratory of Viral Pathogenesis & Infection Prevention and Control (Jinan University), Ministry of Education, Guangzhou, China; ^5^ The First Affiliated Hospital of Jinan University, Guangzhou, China

**Keywords:** NLRP3, inflammasome inhibitor, SARS-CoV-2, Covid-19 biomarker, viral myocarditis

Inflammasome, a multiprotein complex, plays indispensable roles in innate immunity and pathogen infections (Schroder and Tschopp, 2010). It can be activated by pathogen-associated molecular patterns (PAMPs) or damage-associated molecular patterns (DAMPs), including various RNA and DNA viruses, many different types of bacteria and various cellular danger signals (Schroder and Tschopp, 2010; Latz et al., 2013). Activation of inflammasome regulates maturation and secretion of pro-inflammatory cytokines (such as interleukin (IL)-1β and IL-18) and pyroptosis (Latz et al., 2013). NLRP3 (NACHT, LRR and PYD domains-containing protein 3*)* inflammasome is the most extensively concerned and studied inflammasome, which consists of three main components: sensor protein N*LRP3*, adaptor protein ASC (apoptosis-associated speck-like protein containing a CARD), and effecter protein Caspase-1 (Latz et al., 2013). In recent years, many studies have been reported that many different viruses could regulated inflammasome activation by direct mechanisms (the proteins or nucleic acids of viruses could directly affect inflammasome activation) or indirect mechanisms (viruses could affect inflammasome activation by regulating other signal pathways or other proteins). Previous studies have indicated inflammasome participates in viral infections (Lupfer et al., 2015; Zheng et al., 2023). However, what roles that inflammasome plays in the infection of specific virus remain unknown.

In this Research Topic, there are six articles (three original research articles and three review articles) published, focusing on the role of inflammasome in viral infection.

Severe acute respiratory syndrome coronavirus 2 (SARS-CoV-2) infection causes coronavirus disease 2019 (COVID-19) in recent years, and it could activate NLRP3 inflammasome (Pan et al., 2021). Li et al. found that SARS-CoV-2 infection reduces the expression of Bood POZ-containing gene type 2 (BPOZ-2), a scaffold protein for the E3 ubiquitin ligase Cullin 3. BPOZ-2 could interact with NLRP3, mediated its degradation, and negatively regulated NLRP3 inflammasome activation. They suggested that SARS-CoV-2 infection reduced the expression of BPOZ-2 and further promoted NLRP3 inflammasome activation, which likely contributed to SARS-CoV-2-induced hyperinflammation. Hadad et al. found that Capsase-1, ASC, IL-1β and IL-18 were elevated in the plasma of acute COVID-19 patients, and they were still elevated two months after the recovery of infection. Furthermore, they plotted ROC curves for each protein using data from health controls (control) and patients with an active SARS-CoV-2 infection (positive), and indicated Caspase-1, ASC, IL-1β and IL-18 are reliable biomarkers of COVID-19. These findings suggested that inflammasome signaling pathway related proteins could be used to reliably monitor the inflammatory innate immune response in COVID-19 patients. Ou et al. found the differentially expressed genes of severe COVID-19 screened from GSE151764 and GSE183533 by comprehensive transcriptome meta-analysis. Five genes (AXL, MKI67, CDKN3, BCL2 and PTGS2) were found by protein-protein interaction networks and functional analyses. Five genes related to inflammasome could be used as potential markers to identify severe COVID- 19 patients. Diarimalala et al. summarized the roles of NLRP3 inflammasome and other inflammasomes (NLRP1, absent in melanome-2 (AIM-2), Caspase-4 and Caspase-8) during SARS-CoV-2 infection, and also highlighted inflammasome-related inhibitors (including NLRP3 and Gasdermin D inhibitors). This information would be helpful in the understanding of inflammasome roles during SARS-CoV-2 infection and in development of novel therapeutic molecules agaist COVID-19.

Wu et al. systemically summarized basic concepts and detailed content of different types of inflammasomes (including NLRP1, NLRP3, NLRP6, NLRC4, AIM2 and IFI16 inflammasomes), discussed the relationship between viruses (including SARS-CoV-2, influenza virus, human immunodeficiency virus type 1 and hepatitis B virus) and inflammasomes, and reviewed the development of clinical therapies targeting inflammasomes. They also presented that virus-induced inflammasome activation and inflammatory factors could eliminate virus-infected cells by pyroptosis, and that abnormal regulation of inflammasome and pro-inflammatory cytokines contributed to the occurrence of chronic inflammation, thereby driving viral replication. Collectively, they indicated that studying the complex interplay between inflammasome activation and viral infection would enhance the understanding of the pathological mechanisms of corresponding diseases and supply better strategies of treatment.

Viral myocarditis (VMC), characterized by inflammation induced by viral infection, is a life-threatening disease related with dilated cardiomyopathy or heart failure. Xu et al. summarized the detail regulation of the four inflammasomes (NLRP3, Caspase recruitment domain-containing protein 8 (CARD8), Caspase-11 and AIM2) in VMC, the effects of the inflammasome activation in VMC, the roles of inflammasomes in COVID-19-related myocarditis, and the potential therapies (Anakinra, Canakinumab, MCC950, Colchicine, Interleukin-37 and Interferons) targeting inflammasomes or related pathways. They specially emphasized that IL-1β participated in multiple cellular events in both infectious and post-infectious phases, and that inflammasomes could regulate the VMC development by the secretion of IL-1β. Collectively, they explained that the roles of inflammasomes (mainly the NLRP3 inflammasome) in VMC, and predicted several potential therapeutic stretagies targeting inflammasome signaling.

These papers collected by this Research Topic provide not only novel findings but also an up-to-date summary for understanding the roles of inflammasome in viral infections. We also suggest that it is worth conducting more specific studies on the roles of inflammasome in newly breaking-out viral diseases such as the infections of SARS-CoV-2, Monkeypox virus, Dengue virus and so on. In a word, studying the role of inflammasome in viral infections will help to understanding pathogenesis and provide therapeutic strategies for viral infection-related diseases.

## Author contributions

PW: Writing – original draft. YZ: Writing – original draft. PP: Writing – review & editing. YL: Writing – review & editing.
